# Neurocognitive Improvements in Lithium-Treated Older Adult Bipolar Patients on Antipsychotics

**DOI:** 10.1192/j.eurpsy.2026.11209

**Published:** 2026-07-31

**Authors:** K. Diakaki, E. M. Tsapakis, G. Zioris, A. Konsta, K. N. Fountoulakis

**Affiliations:** 1 3rd Department of Psychiatry, Aristotle University of Thessaloniki, Thessaloniki; 2 Department of Psychiatry, University General Hospital; 3 Agios Charalambos, Mental Health Clinic, Heraklion, Crete; 4 Department of Psychiatry; 5 1st Department of Psychiatry, Aristotle University of Thessaloniki, Thessaloniki, Greece

## Abstract

**Introduction:**

Cognitive dysfunction in bipolar disorder (BD) affects memory, attention, and executive function, and has been shown to persist in euthymia. Lithium remains the gold standard for the treatment of mania and for relapse prevention and has gained particular recognition despite concerns about neurotoxicity at high doses and other less common adverse effects. Further, lithium has also demonstrated neuroprotective properties and shown potential benefits in neurodegenerative conditions.

**Objectives:**

Lithium has been proposed to exert neuroprotective effects, yet its relationship to cognitive performance remains unclear. This study aimed to compare neurocognitive performance in BD patients on antipsychotics grouped by long-term lithium use.

**Methods:**

We recruited older adult BD (OABD) out-patients in a cross-sectional opportunistic design to complete a comprehensive neurocognitive battery standardized for the Greek population. We used IBM SPSS v28.0 for analysis. Participants were divided into current lithium-treated (n = 19) and non-lithium-treated (n = 21) groups. Performance was compared using Z-scores, Bonferroni corrected at p < 7.6×10^-4^.

**Results:**

Forty OABD patients, with a mean age of 58.3 (5.89) years, 15 (37.5%) male, 95% on antipsychotics entered the study. Lithium-treated patients demonstrated significantly better cognitive performance in attention span and verbal memory [higher scores on Digit Span Forward (Z = 1.43 vs. 0.17; p = 3.6×10^-5^), Digit Span Backward (Z = 0.71 vs. –0.74; p = 2.4×10^-5^), and Stories Immediate and Delayed Recall (Z = 0.02 vs. –1.05 and 0.32 vs. –0.98; p < 1×10^-5^)], and in recognition memory [RAVLT Recognition (Z = 1.14 vs. –1.43; p = 1.4×10^-6^)]. The largest difference emerged in processing speed and visual attention [higher scores on Symbol Digit Modalities Test (Z = 0.42 vs. –1.51; p = 4.7×10^-8^)]. Verbal fluency and executive control were also superior on COWAT (Z = 0.16 vs. –0.99; p = 6.3×10^-5^). No significant associations were found between cognitive outcomes and illness duration, episode frequency, or medication load.

**Conclusions:**

Lithium use was associated with cognitive advantages across memory, attention, and processing speed. Our findings provide support for the role of lithium in cognitive preservation and are worthy of further investigation in larger longitudinal studies.

**Disclosure of Interest:**

None Declared

